# Progression of Subclinical Hypothyroidism in a Mexican Public Hospital Population: A Retrospective Cohort Study

**DOI:** 10.7759/cureus.82923

**Published:** 2025-04-24

**Authors:** Rafael Violante-Ortiz, Norma Fernández-Ordóñez, Emanuel Narvaez Gallifa, Erick E Hernandez Molina, Jose E Guerra Cardenas, Elizabeth Reyna-Beltrán, Dylan Castillo Hernández, Luis S Díaz Martínez, Izmene N Badillo Grijalva, Jaidy M Bautista Sánchez

**Affiliations:** 1 Endocrinology and Metabolism, Facultad de Medicina de Tampico “Dr. Alberto Romo Caballero” Universidad Autónoma de Tamaulipas, Tampico, MEX; 2 Endocrinology and Metabolism, Centro de Estudios de Investigación Metabólicos y Cardiovasculares, S.C., Madero, MEX; 3 Obstetrics and Gynecology, Facultad de Medicina de Tampico “Dr. Alberto Romo Caballero” Universidad Autónoma de Tamaulipas, Tampico, MEX; 4 Biological Sciences, Facultad de Medicina de Tampico “Dr. Alberto Romo Caballero” Universidad Autónoma de Tamaulipas, Tampico, MEX

**Keywords:** clinical endocrinology, subclinical hypothyroidism (sch), thyroid peroxidase antibodies (tpoab), thyroid-stimulating hormone (tsh), tsh levels

## Abstract

Background and aim: Treatment of subclinical hypothyroidism (SCH) is controversial. The uncertainty of the levels of thyroid-stimulating hormone (TSH) that warrant treatment and the risk of progression to overt hypothyroidism may lead to overtreatment. This study aimed to assess the persistence of SCH and its short-term progression to overt hypothyroidism in patients referred to an outpatient endocrinology clinic in Southern Tamaulipas, Mexico, and to identify predictive factors for progression to overt hypothyroidism.

Methods: This analytic, observational, and retrospective study analyzed records from 1100 patients at a Mexican public hospital between 2018 and 2019. Exclusion criteria included prior hypothyroidism, levothyroxine use, pregnancy, TSH ≥10.0 mIU/L, age <18 years, and non-completion of follow-up. A final sample of 222 patients with SCH (defined as TSH >4.2 and <10.0 mIU/L, with normal T4 levels) was followed for three months, assessing the regression, persistence, and progression of TSH levels. Statistical analyses included a chi-squared test and Student’s t-test. Statistical significance was set at alpha=0.05.

Results: The study included 181 (81.5%) females with a mean age of 49.7 years (±13.5). After three months, 158 (71.2%) patients regressed to euthyroidism, 47 (21.2%) remained subclinically hypothyroid, and 17 (7.6%) progressed to overt hypothyroidism. The highest progression rate to overt hypothyroidism (16.0%) was observed in patients with initial TSH levels >6.0 to ≤8.0 mIU/L (relative risk: 5.4; 95% confidence interval {CI}: 1.83-16.0, p<0.001). Mean baseline TSH levels were 6.57±1.11 mIU/L (95% CI: 6.00-7.15, p<0.001) in those who progressed to overt hypothyroidism. No association was observed between symptoms and disease progression.

Conclusion: Most patients with SCH regressed to an euthyroid state without treatment in the short term, supporting a monitoring-first approach.

## Introduction

Subclinical hypothyroidism is defined by an elevated thyroid-stimulating hormone (TSH) level with normal serum free thyroxine (T4), making its diagnosis dependent on laboratory findings. Although some patients may present with mild, non-specific symptoms such as fatigue or constipation, these symptoms are not reliable indicators of subclinical hypothyroidism. Therefore, laboratory testing remains the definitive method for diagnosis. In Mexico, national data on thyroid disorders are very limited, leading to an overreliance on information that might underestimate or overestimate the burden of thyroid disease. The prevalence of subclinical hypothyroidism in Mexico, based on independent research groups, is estimated to range from 2.9% to 8.3% [1-3]. This variation may be attributed to the mean age of study participants and the cutoff TSH value used in those studies. In the United States, the Third National Health and Nutrition Examination Survey (NHANES III) reported a prevalence of 4.3% [4], although more recent studies indicate lower rates due to variations in study methodology [5]. The condition is more prevalent in women, increases with age, and occurs less frequently in Mexican Americans compared to White individuals [4].

The etiology of subclinical hypothyroidism mirrors that of overt hypothyroidism, with chronic lymphocytic thyroiditis, including Hashimoto's and atrophic thyroiditis, being the most common causes [6]. Other etiologies include thyroid tissue damage due to treatments such as radioactive iodine or external radiation therapy [6]. The majority of patients with subclinical hypothyroidism present with TSH levels below 10.0 mIU/L and remain asymptomatic. However, identifying symptomatic patients can be challenging due to the non-specific nature of their symptoms [7].

The annual risk of progression from subclinical hypothyroidism to overt hypothyroidism ranges from 2% to 6% [8-10]. This risk is elevated among women, individuals with higher thyrotropin levels, those with increased serum thyroid peroxidase antibodies (TPO-Ab), and those exhibiting low-normal free T4 levels [8-10]. Patients who test positive for serum TPO-Ab are twice as likely to develop overt hypothyroidism compared to those without antibodies [8,9,11-13]. A thyrotropin level of ≥10.0 mIU/L has an even stronger association with the progression to overt hypothyroidism and is the value most widely accepted to determine whether to treat patients with subclinical hypothyroidism [10]. Among individuals with a single elevated thyrotropin measurement of less than 7 mIU/L, up to 46% may see their thyrotropin levels normalize within two years [9,11,14].

Current recommendations for the management of this condition exist; however, the treatment of patients with TSH values between 4.5 and 10.0 mIU/L is controversial. This can lead to unnecessary treatment, which may persist for several years due to the challenges associated with discontinuing thyroid hormone therapy once it has been initiated.

The purpose of this study was to assess the persistence of subclinical hypothyroidism and its short-term progression to overt hypothyroidism among patients referred to the outpatient endocrinology clinic at IMSS Hospital Regional General No. 6 in Madero City, Tamaulipas, Mexico, as well as to evaluate the factors most predictive of progression to overt hypothyroidism.

## Materials and methods

This analytic, observational, and retrospective study analyzed records from 1100 patients referred from primary care to the Outpatient Endocrinology Department at Instituto Mexicano del Seguro Social Hospital General Regional No. 6 in Madero City, Tamaulipas, Mexico, between January 2018 and January 2019 due to suspected hypothyroidism; with a three-month follow-up period to assess outcomes.

Inclusion and exclusion criteria

Exclusion criteria included a prior diagnosis of hypothyroidism, ongoing levothyroxine treatment, pregnancy, TSH levels ≥10.0 mIU/L, age under 18 years, and failure to attend a second follow-up appointment. Patients diagnosed with subclinical hypothyroidism, defined as a thyroid-stimulating hormone (TSH) level greater than 4.2 mIU/L and up to 10.0 mIU/L with normal free thyroxine (T4) levels, were included in the study. A total of 222 individuals met these criteria and were selected for final analysis.

Data collection

Baseline data were extracted from electronic medical records, including demographic variables such as age, sex, height, and weight. Symptoms reported at the initial consultation, including fatigue, hair loss, dry skin, and a combination of fatigue with hair loss, were also documented. Laboratory parameters analyzed included TSH, free T4 (FT4), free triiodothyronine (FT3), total T4, and total T3.

**Figure 1:**
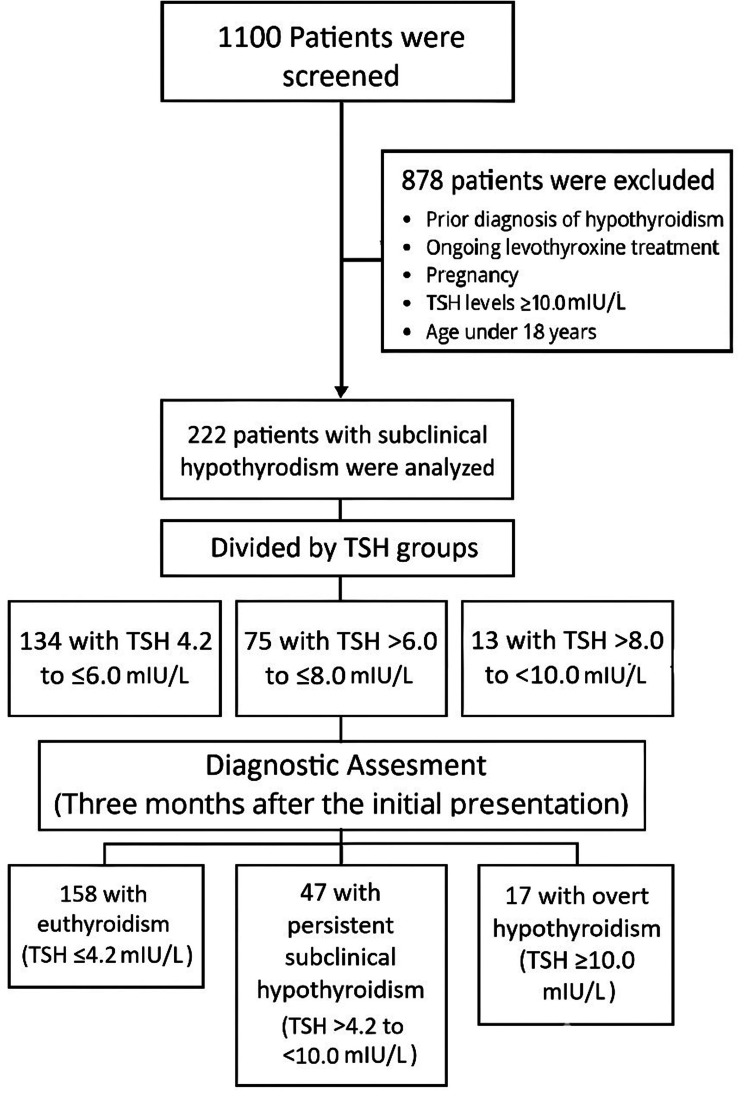
Screening, baseline TSH group organization, and follow-up with diagnostic assessment. TSH: thyroid-stimulating hormone

Patients were categorized into the following three baseline TSH groups: 4.2 to ≤6.0 mIU/L, with 134 patients (60.4%); >6.0 to ≤8.0 mIU/L, with 75 patients (33.8%); and >8.0 to <10.0 mIU/L, with 13 patients (5.8%). At the three-month follow-up, a second thyroid function test was performed, and patients were categorized into three outcome groups as follows: group A, with 158 patients (71.2%) who regressed to euthyroidism (TSH ≤4.2 mIU/L); group B, with 47 patients (21.2%) who remained subclinically hypothyroid (TSH >4.2 to <10.0 mIU/L); and group C, with 17 patients (7.6%) who progressed to overt hypothyroidism (TSH ≥10.0 mIU/L). None of the patients received treatment throughout the study period. Figure 1 provides a visual representation of the screening process, baseline TSH group organization, and follow-up with diagnostic assessment.

Statistical analyses

Statistical analyses were conducted using SPSS version 26 (Armonk, NY: IBM Corp.). Normally distributed continuous data were expressed as means±standard deviation (SD) and were compared using Student's t-test. Categorical variables were presented as frequencies and percentages and analyzed using the chi-squared test. The relative risk (RR) and 95% CI were reported for each predictor. Statistical significance was set at alpha=0.05.

## Results

The baseline characteristics of the sample included a mean age of 49.7 years (±13.5), a mean height of 1.61 (±0.05) meters, and a mean weight of 68.9 (±6.8) kg. The sample comprised 181 females (81.5%). The age range of 18 to 59 years predominated, with 158 patients (71.2%). Table 1 compares the first and second visit thyroid function test levels, with a significant difference observed only in TSH levels (p<0.001).

**Table 1:** First and second visit thyroid function tests levels. FT4: free thyroxine; FT3: free triiodothyronine; TSH: thyroid-stimulating hormone

Thyroid function test	First visit, n=222 (mean±SD)	Second visit, n=222 (mean±SD)	p-Value
FT4	1.31±0.31	1.32±0.32	0.641
FT3	2.84±0.72	3.31±3.2	0.059
Total T4	8.05±1.7	8.55±2.1	0.975
Total T3	1.27±0.82	1.24±0.71	0.664
TSH	5.83±1.1	4.38±4.3	<0.001

**Figure 2:**
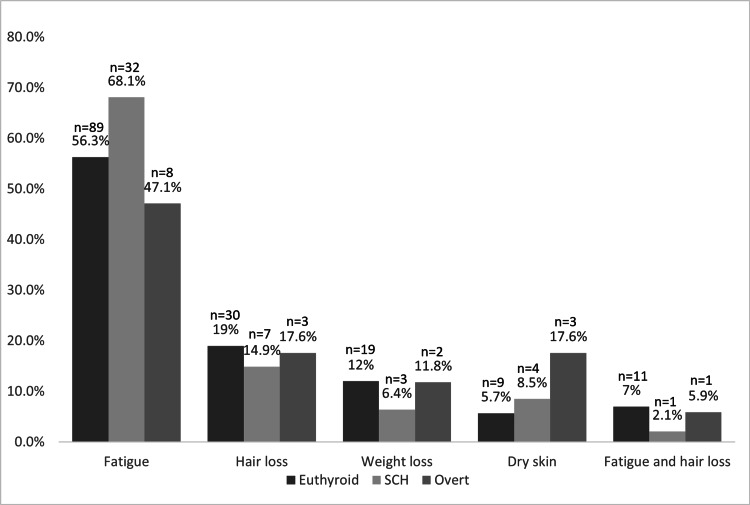
Relationship of symptoms and final thyroid diagnosis at the second visit. SCH: subclinical hypothyroidism

Figure 2 illustrates the relationship between symptoms and final thyroid diagnosis at the second visit (three months after the initial presentation). In the group that progressed to overt hypothyroidism, fatigue was the most common symptom in eight patients (47.1%), followed by hair loss in three patients (17.6%), and dry skin in three patients (17.6%). No significant relationship was found between symptoms and progression to overt hypothyroidism (p=0.527).

**Figure 3:**
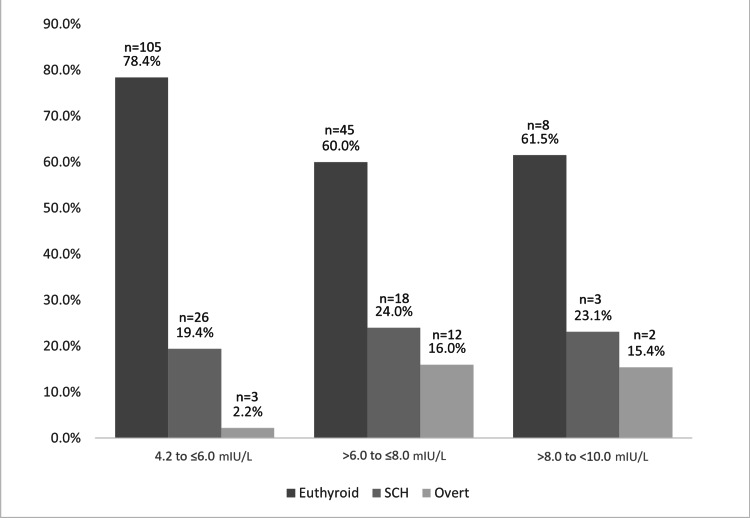
Relationship between baseline TSH levels by group and their changes at follow-up. SCH: subclinical hypothyroidism; TSH: thyroid-stimulating hormone

Thyroid function follow-up over three months revealed that 158 (71.2%) of patients showed regression and were classified as euthyroid, 47 (21.2%) remained subclinically hypothyroid, and 17 (7.6%) progressed to overt hypothyroidism. The mean baseline TSH levels were 5.68±1.06 mIU/L (95% CI: 5.52-5.85; p<0.001) in patients who normalized to euthyroid, 6.15±1.14 mIU/L (95% CI: 5.82-6.49; p<0.001) in those who remained with subclinical hypothyroidism, and 6.57±1.11 mIU/L (95% CI: 6.00-7.15; p<0.001) in those who progressed to overt hypothyroidism. Table 2 shows first and second visits thyroid function tests levels according to their final thyroid diagnosis group. Among 134 patients with baseline TSH levels between 4.2 and ≤6.0 mIU/L, 105 (78.4%) normalized their TSH levels (p<0.001). Three patients with TSH levels between 4.2 and ≤6.0 mIU/L progressed to overt hypothyroidism, representing a progression rate of 2.2% (relative risk {RR}: 7.1; 95% CI: 2.1-24.01; p<0.001). Twelve patients with levels between >6.0 and ≤8.0 mIU/L progressed, representing a progression rate of 16.0% (RR: 5.4; 95% CI: 1.83-16.00; p<0.001). Two patients with levels between >8.0 and <10.0 mIU/L progressed, representing a progression rate of 15.4% (RR: 2.3; 95% CI: 0.47-11.60; p<0.001). Figure 3 illustrates the relationship between baseline TSH levels by group and their changes at follow-up. No statistically significant differences were observed for levels between >8.0 and <10.0 mIU/L.

**Table 2:** First and second visit thyroid function tests levels according to their final thyroid diagnosis group. *First visit. **Second visit. FT4: free T4; FT3: free T3; T4: total T4; T3: total T3; TSH: thyroid-stimulating hormone

Thyroid profile	Euthyroid, n=158 (A)	SCH, n=47 (B)	Overt, n=17 (C)	A vs. B	A vs. C	B vs. C
FT4*	1.32±0.33	1.31±0.28	1.24±0.30	0.868	0.328	0.369
FT3*	2.83±0.66	2.78±0.93	3.32±0.70	0.767	0.119	0.244
T4*	8.09±1.8	7.92±0.94	8.06±2.3	0.702	0.971	0.839
T3*	1.27±0.82	1.17±0.28	1.70±0.86	0.637	0.264	0.026
TSH*	5.68±1.06	6.09±1.3	6.57±1.11	0.029	0.001	0.176
FT4**	1.34±0.32	1.32±0.28	1.01±0.35	0.720	0.004	0.011
FT3**	3.43±3.7	3.16±0.86	2.84±0.59	0.798	0.542	0.251
T4**	8.86±2.2	8.15±1.3	7.21±1.9	0.268	0.009	0.165
T3**	1.25±0.81	1.30±0.34	1.21±0.36	0.814	0.742	0.467
TSH**	2.0±1.1	8.17±1.6	15.39±4.2	<0.001	<0.001	<0.001

Table 3 compares the baseline demographics and clinical characteristics by final thyroid diagnosis group. In general, all three groups shared similarities, including an older mean age, a predominance of females, and a higher proportion of individuals aged 18-59 years. Group comparisons revealed that group A vs. group B showed significant differences in baseline characteristics for sex (p<0.001), height (1.61±0.05 vs. 1.65±0.06 m, p<0.001), and weight (68.0±6.3 vs. 71.6±8.0 kg, p=0.004). At the second visit, there were significant differences in weight (68.2±6.4 vs. 71.8±8.1 kg, p=0.005) and TSH levels (2.0±1.1 vs. 8.17±1.6 mIU/L, p<0.001).

**Table 3:** Baseline demographics and clinical characteristics by final thyroid diagnosis group. *Second visit. SCH: subclinical hypothyroidism

Baseline characteristic	Euthyroid, n=158 (A)	SCH, n=47 (B)	Overt, n=17 (C)	A vs. B	A vs. C	B vs. C
Age (years)	51.0±13.6	45.8±12.9	49.3±13.8	0.056	0.879	0.628
Female	145 (91.8%)	24 (51.1%)	12 (70.6%)	<0.001	0.051	0.125
Male	13 (8.2%)	23 (48.9%)	5 (29.4%)			
Age 18-59 years	106 (67.1%)	40 (85.1%)	12 (70.6%)	0.044	0.950	0.491
Age ≥60 years	52 (32.9%)	7 (14.9%)	5 (29.4%)			
Height (cm)	1.61±0.05	1.65±0.06	1.63±0.06	<0.001	0.423	0.309
Weight (kg)	68.0±6.3	71.6±8.0	69.7±5.6	0.004	0.577	0.589
Weight (kg)*	68.2±6.4	71.8±8.1	69.7±5.8	0.005	0.671	0.515
BMI (kg/m²)	26.3±2.1	26.3±1.9	26.4±2.0	0.990	NA	0.995

Group A vs. group C showed no significant differences in baseline characteristics for sex, height, or weight. Significant differences were found in TSH levels at both the first (5.68±1.06 vs. 6.57±1.11 mIU/L, p=0.001) and second visits (2.0±1.1 vs. 15.39±4.2 mIU/L, p<0.001). Group B vs. group C showed significant differences only in TSH levels at the second visit (8.17±1.6 vs. 15.39±4.2 mIU/L, p<0.001).

## Discussion

This study described the natural course of subclinical hypothyroidism in 222 patients within a Mexican population and analyzed laboratory and potential clinical prognostic factors for progression to overt hypothyroidism. This may be the first cohort study of subclinical hypothyroidism in Mexico to describe its natural course. Our sample included patients from different regions of Mexico, with the majority coming from the Southern Tamaulipas area.

Seventeen patients (7.6%) progressed to overt hypothyroidism, slightly higher than the annual rate of progression to overt hypothyroidism, which ranges from 2% to 4% [7]. The normalization rate was 158 patients (71.2%), the highest in our study; this spontaneous recovery has been described in cohort studies, although there is wide rate variation between studies [9,14-16]. In one of those studies, the spontaneous normalization rate in a five-year follow-up period was 62.1% among patients with a serum TSH level of >5.5 to ≤10.0 mIU/L, close to the rate seen in our study with very similar TSH range values [14]. The persistent rate of subclinical hypothyroidism was seen in 47 (21.2%) patients in the present study, which is lower than what was found in other studies where this rate ranged between 36.7%, 52.1%, and 68% at five, three, and 9.2 years of follow-up, respectively [9,17,18].

The majority of patients in the present study were women, representing 181 (85.1%), aged 18 to 59 years. This reflects the high prevalence of this thyroid disease among female patients and is similar to other studies where the female population was predominant [15,17].

Regarding the predicting factors of progression to overt hypothyroidism, no association was observed between symptoms at the first visit and progression to overt hypothyroidism at follow-up three months after the initial presentation. Notably, patients with prominent symptoms at baseline normalized, and patients with fewer symptoms at baseline progressed to overt hypothyroidism.

This study observed that patients with baseline TSH levels between 4.2 and ≤6.0 mIU/L exhibited the highest normalization rate at 105 patients (78.4%) among all groups. Interestingly, individuals with initial TSH levels >6.0 to ≤8.0 mIU/L demonstrated the highest progression rate to overt hypothyroidism, 12 patients (16.0%), with a relative risk of 5.4 (95% CI: 1.83-16.00, p=0.001). Despite not having the highest TSH levels, this group also showed the lowest rate of normalization, with only 45 patients (60.0%) returning to normal thyroid function.

The limitations of this study include the absence of serum TPO-Ab measurement, which is an independent risk factor for progression to overt hypothyroidism. Additionally, serum levels of TPO-Ab and TSH tend to follow a parallel course in patients with subclinical hypothyroidism; however, these measurements were not taken due to institutional policies [19,20]. Another limitation is the short follow-up period, as additional long-term changes might have been observed with extended follow-up.

## Conclusions

In conclusion, in this study, most patients' SCH regressed to an euthyroid state without treatment in the short term. This finding supports the feasibility of a monitoring-first approach rather than immediate treatment, potentially avoiding unnecessary and costly medical interventions. Comparatively, these results align with previous studies that report a high rate of spontaneous normalization of TSH in patients with SCH, although our progression rate to overt hypothyroidism was slightly higher than reported in the literature.

It is advisable for clinical practice to lean towards a surveillance protocol, particularly in patients without significant symptoms or clear risk factors for progression. Future research should focus on more extensive longitudinal studies and evaluate the impact of different TSH thresholds for treatment on clinical outcomes.
